# In the works: Patient and public involvement and engagement in healthcare decision‐making

**DOI:** 10.1111/hex.13339

**Published:** 2021-08-21

**Authors:** Bert de Graaff, Tineke Kleinhout‐Vliek, Hester Van de Bovenkamp

**Affiliations:** ^1^ Erasmus School of Health Policy and Management Erasmus University Rotterdam Rotterdam The Netherlands; ^2^ Copernicus Institute of Sustainable Development Utrecht University Utrecht The Netherlands

**Keywords:** healthcare decision‐making, patient and public involvement and engagement, work

1

In the September 2019 Special Issue of Health Expectations on Patient and Public Involvement and Engagement (PPIE) initiatives, Hickey and Chambers^1^ highlighted ‘the importance of […] promoting PPIE across all areas of health and social care both nationally and internationally’ (p. 607). This resonates with the widespread recognition of PPIE's potential to improve the quality of decision‐making and increase fairness, responsiveness and legitimacy. Such a positive attitude towards the instrumental and ideological values of involvement often lends support to the conclusion that patients should always be encouraged to climb Arnstein's famous ladder of participation: the more, the merrier. Reflecting on the Special Issue's contents, Hickey and Chambers do note a considerable gap between ideal and practice. Indeed, many authors have described the complexities surrounding the implementation of PPIE. These complexities include limited attention of professionals, policymakers and researchers, a lack of enthusiasm among patients and citizens, issues around the representativeness of those who participate and lack of clarity about the results.

We propose that it is time to move beyond discussing PPIE as something that we can never have enough of and to start examining more thoroughly the *work* necessary to make PPIE work in healthcare decision‐making. In organizational studies and elsewhere, the ‘turn to work’ has emphasized the need to consider the situated and shared effort, purposeful and strategic, of people to affect their social–symbolic context.^2^ Hickey and Chambers themselves advocate an appreciation of PPIE as coproduction. They write that such a view of PPIE entails careful and inclusive relationship development wherein all those involved are respected and where all contributions are valued (1). We agree that PPIE requires an inclusive approach and that initiating and sustaining PPIE initiatives require considerable effort. However, we deem it necessary to take it one step further. We hold that uncritical promotion of PPIE distracts from the highly situated and contextualized efforts needed to make PPIE work. In this sense, such promotion risks actively thwarting promising attempts to ‘bridge the gap’. Concretely, it may undermine the PPIE values and goals as patients' input is stymied by the mould in which it is poured.^3^

We propose that considering PPIE not only as coproduction but also as work means that each PPIE initiative should be tailor‐made in terms of whom to involve, how to involve them and how to value their contributions. A short note on each is presented here.

Whom to involve? The outcomes of PPIE initiatives strongly depend on who is involved or engaged or the way in which they are being represented. The type of work required in many PPIE initiatives appears to suit primarily highly educated, or even ‘professionalized’, people^4^; reaching and involving more of them might not necessarily bring more quality to a decision‐making process. Questions for reflection and empirical research include how to increase the diversity of participants in PPIE initiatives and how to reach a specific group.

How to involve patients and the public? In any PPIE initiative as elsewhere, the method used to involve diverse perspectives actively shapes ‘the patient’ and ‘the public’ that is being attended to. The method is thus not a neutral element. As PPIE is situated and contextualized work, the method's effect largely depends on the specifics of the situation. We suggest that this means that methodologies of participation require calibration and realignment on the way. It evokes questions such as the following: What kind of public does this method produce here and does this match our goal(s)? What kind of public(s) are left out?

How to value their contributions? Often, rational, argumentative ways of participation have won out over emotional ones, for one pushing back experiential wisdom in favour of more readily objectified contributions, this even though emotions contain powerful, convincing, predictive and sometimes good (and sometimes malicious) drivers for decision‐making processes. Emotions are deemed necessary for practical and moral decision‐making and may thus serve as a ‘beneficial guide’ in PPIE initiatives.^5^ How to value all types of contributions but emotive ones, in particular, offers food for thought. Storytelling and narrative analyses might help.

Conceptualizing PPIE as work helps us explain the gap between ideal and practice; it helps us move away from linear and generalist ‘ladder’ approaches to PPIE. Instead, it encourages us to consider the practical work needed for PPIE initiatives. Careful consideration of whom to involve, how to involve them and how to value their contributions should inform this. Hopefully, this will result in tailor‐made PPIE initiatives in terms of matched processes and goals, which are arrived at through careful reflection and resource use. Specifically, we encourage thinking and empirical research on how to increase the diversity of perspectives, recalibrate methodologies and value emotions. These elements appear to have been underexposed and underutilized so far.

## Data Availability

Data and the original report (in Dutch) that support this letter are available from the corresponding author upon reasonable request.
